# Potential benefits of mesenchymal stem cells and electroacupuncture on the trophic factors associated with neurogenesis in mice with ischemic stroke

**DOI:** 10.1038/s41598-018-20481-3

**Published:** 2018-02-01

**Authors:** Yu Ri Kim, Sung Min Ahn, Malk Eun Pak, Hong Ju Lee, Da Hee Jung, Yong-Il Shin, Hwa Kyoung Shin, Byung Tae Choi

**Affiliations:** 1 Department of Korean Medical Science, School of Korean Medicine, Pusan National University, Yangsan, 50612 Republic of Korea; 2 Korean Medical Science Research Center for Healthy Aging, Pusan National University, Yangsan, 50612 Republic of Korea; 3 Graduate Training Program of Korean Medicine for Healthy Aging, School of Korean Medicine, Pusan National University, Yangsan, 50612 Republic of Korea; 4 Department of Rehabilitation Medicine, School of Medicine, Pusan National University, Yangsan, 50612 Republic of Korea; 5 Division of Meridian and Structural Medicine, School of Korean Medicine, Pusan National University, Yangsan, 50612 Republic of Korea

## Abstract

The beneficial effects of mesenchymal stem cells (MSCs) and electroacupuncture (EA) on neurogenesis and related trophic factors remain unclear. Bone marrow MSCs (mBMSC) were transplanted into the striatum of mice with middle cerebral artery occlusion (MCAO), and EA stimulation was applied at two acupoints, Baihui and Dazhui. EA treatment significantly improved motor function, and a synergistic effect of combined mBMSC and EA treatment was observed. Combined mBMSC and EA treatment reduced prominent atrophic changes in the striatum and led to proliferation of neural progenitor cells in the subventricular zone (SVZ) and the surrounding areas of the striatum (SVZ + striatum) of MCAO mice. The mBMSC and EA treatment markedly enhanced mature brain-derived neurotrophic factor (mBDNF) expression in the SVZ + striatum and hippocampus of mice with MCAO, and combined treatment enhanced neurotrophin-4 (NT4) expression. The number of mBDNF- and NT4-positive neurons in the SVZ + striatum and hippocampus increased following EA treatment. Combined treatment led to an increase in the expression levels of phosphorylated cAMP response element binding protein in the neuroblasts of the striatum. Our results indicate that combined MSC and EA treatment may lead to a better therapeutic effect via co-regulation of neurotrophic factors in the brain, by regulating neurogenesis more than single therapy.

## Introduction

Endogenous neurogenesis is restricted in the adult mammalian brain; however, brain injury in pathologies such as stroke elicits a latent neurogenic program^1^. Successful neurogenesis in the brain provides an attractive therapeutic prospect for a variety of neurodegenerative diseases^2,3^. This self-renewal and multilineage differentiation from neuronal progenitor cells is regulated by a specific set of signals that comprises both cell-intrinsic programs and extrinsic factors^4,5^. Extrinsic factors influencing the stem cell niche allow trophic factors to persist and regulate the proliferation and differentiation of the progenitor cell population^3–6^.

Mesenchymal stem cell (MSC) transplantation is considered a treatment for cerebral ischemia, because MSCs can be readily obtained from the patient and easily cultured *in vitro*, thereby leading to weak immunological reactions and improved safety^7–10^. Moreover, one hypothetical explanation for the efficacy of MSC transplantation is based on the trophic effect, which is mediated by various kinds of trophic factors produced by the MSCs themselves^11–13^. MSCs can secrete a variety of trophic factors with autocrine and paracrine modes of action under hypoxic or ischemic conditions^14–16^. MSCs also induce parenchymal cells in the brain to secrete endogenous trophic factors to promote functional recovery after stroke^9,16,17^. MSC transplantation has been shown to have a nursing function to promote neurogenesis in animal models with cerebral ischemia^13,18^. Finally, MSCs can also mediate and promote neurogenesis, and ameliorate neurological functional deficits, due to their ability to produce growth-promoting factors^11,14,16,19^.

Electroacupuncture (EA) is a modern type of traditional acupuncture that involves inserting needles and providing stimulation through the application of electric pulses instead of hand manipulation. EA has been used in patients with stroke and for post-stroke rehabilitation, as it can improve neurological impairments with no serious adverse events^20,21^. Electrical stimulation activates several classes of sensory fibers as well as some molecules, including trophic factors, that are produced as a mediator of acupuncture effects in the central nervous system^6,22^. EA treatment has a therapeutic effect on neurological functional recovery and brain injury related to cerebral ischemic insult through the enhancement of endogenous neurogenesis via trophic factors^20,23–25^.

MSC transplantation and EA stimulation are prospective therapies for ischemic stroke that are administered to improve neurological deficits while reducing brain injury. The mechanisms underlying these therapeutic effects involve enhancing endogenous neurogenesis through the activation of trophic factors^14,20^. Therefore, we hypothesized that MSC and EA treatment may stimulate the expression of trophic factors in the brain, consequently leading to the recovery of neurological function. We aimed to examine the effects of mBMSC and EA treatment on neurological function after ischemic brain injury, by investigating endogenous neurogenesis with a focus on trophic factors.

## Results

### Effects of mBMSC and EA treatment on post-stroke behavior

**Figure 1:**
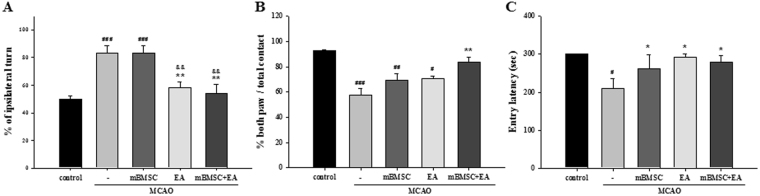
Behavioral tests for motor and cognitive dysfunctions. (**A**) Corner test (n = 6–7). (**B**) Cylinder test (n = 5). (**C**) Passive avoidance test (n = 5). Mice with MCAO treated with EA showed significant recovery of sensorimotor function with spatial memory, compared to mice with MCAO treated with vehicle. Combined mBMSC and EA treatment led to higher improvements in the motor function of both paws than vehicle treatment. Mean ± SEM. ^#^*p* < 0.05, ^##^*p* < 0.01, and ^###^*p* < 0.001 versus control group; ^*^p < 0.05 and ^**^p < 0.01 versus vehicle-treated MCAO group; ^&&^*p* < 0.01 versus mBMSC-treated MCAO group.

The corner test revealed that the number of turns to the ipsilateral side was significantly higher in MCAO mice treated with either vehicle or mBMSC alone than in control mice. However, this number was significantly lower in the EA treatment group [Fig. 1A, F_(4,25)_ = 11.647, *p* < 0.001]. The cylinder test revealed that the number of times the mice held on to the transparent acrylic cylinder wall with both paws was lower in MCAO mice treated with vehicle, mBMSC, or EA than in control mice; combined mBMSC and EA treatment, however, led to a significant increase in this number [Fig. 1B, F_(4,20)_ = 11.420, *p* < 0.001]. The entry latency in the passive avoidance test was lower in mice with MCAO than in control mice. We found a significantly higher entry latency in MCAO mice treated with mBMSC and/or EA than in vehicle-treated MCAO mice [Fig. 1C, F_(4,20)_ = 3.700, *p* = 0.013]. These findings suggest that EA treatment after MCAO, especially when combined with mBMSC treatment, improves sensorimotor function and restores motor function in both paws.

### Effects of mBMSC and EA treatment on brain atrophy

**Figure 2:**
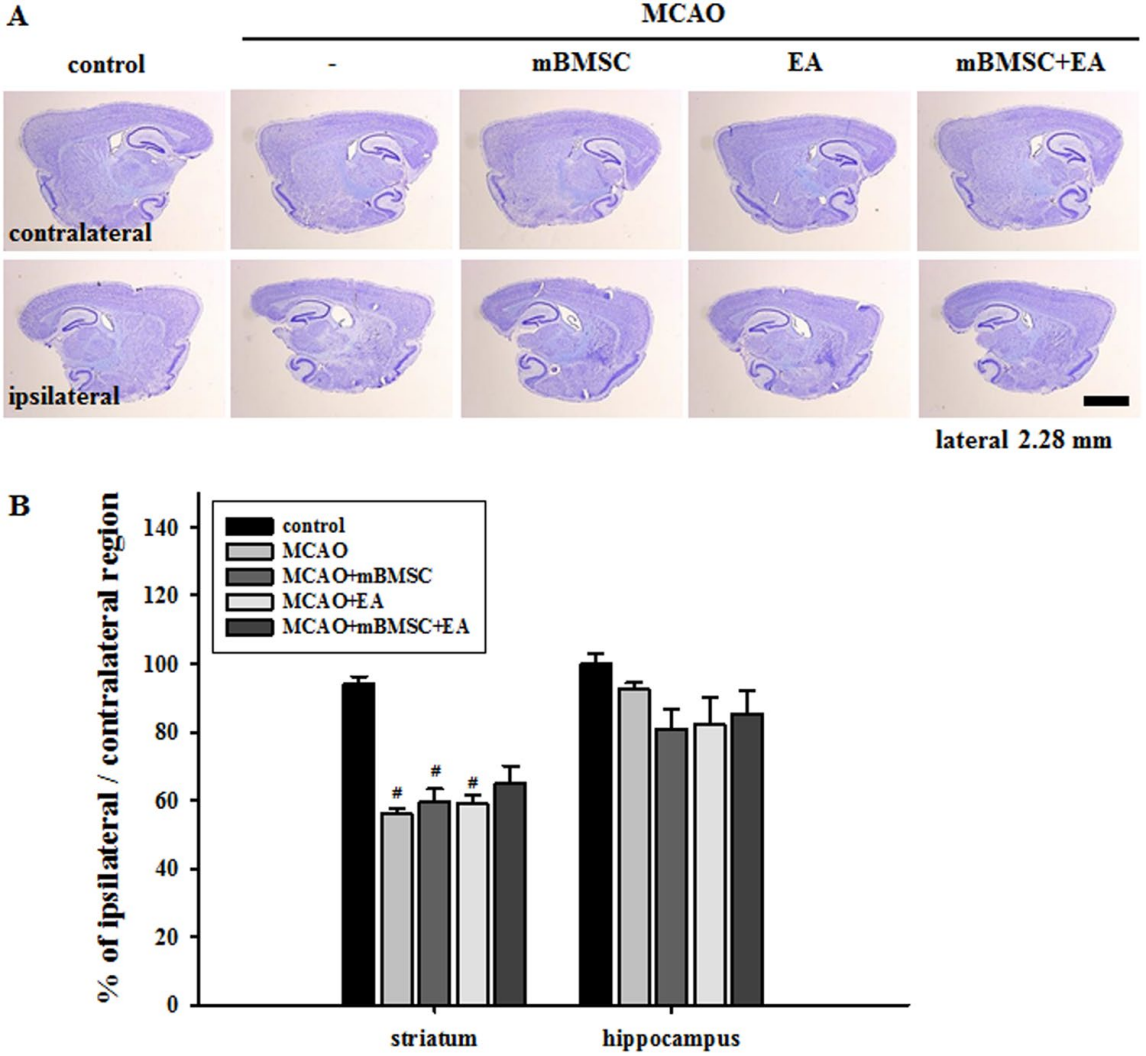
Comparison of atrophic volumes in the striatum and hippocampus. (**A**) Photomicrograph (cresyl violet stain) showing the striatum and hippocampus and (**B**) histogram of the histological analysis (n = 6). The combined mBMSC and EA treatment group showed lesser atrophy in the striatum than the other MCAO groups. ^#^*p* < 0.05 versus control group. Scale bar = 1 mm.

In order to investigate the effects of mBMSC and EA treatment on histological changes in the brain, we analyzed atrophic changes in the striatum (the site of primary damage related to MCAO and the site of mBMSC transplantation) and hippocampus (the site showing cell death related to MCAO). We observed marked atrophic changes in the striatum of MCAO mice treated with vehicle, mBMSC, or EA alone than in the striatum of control mice [F_(4,20)_ = 31.124, *p* < 0.001]; combined treatment with mBMSC and EA somewhat restored the atrophy of brain in the striatum, but it was not significant. We found no significant differences in the atrophy levels in the hippocampus among the groups (Fig. 2). These results suggest that combined treatment with mBMSC and EA can alleviate atrophic changes in the striatum, the primary site damaged by ischemia.

### Effects of mBMSC and EA treatment on neurogenesis in the brain

**Figure 3:**
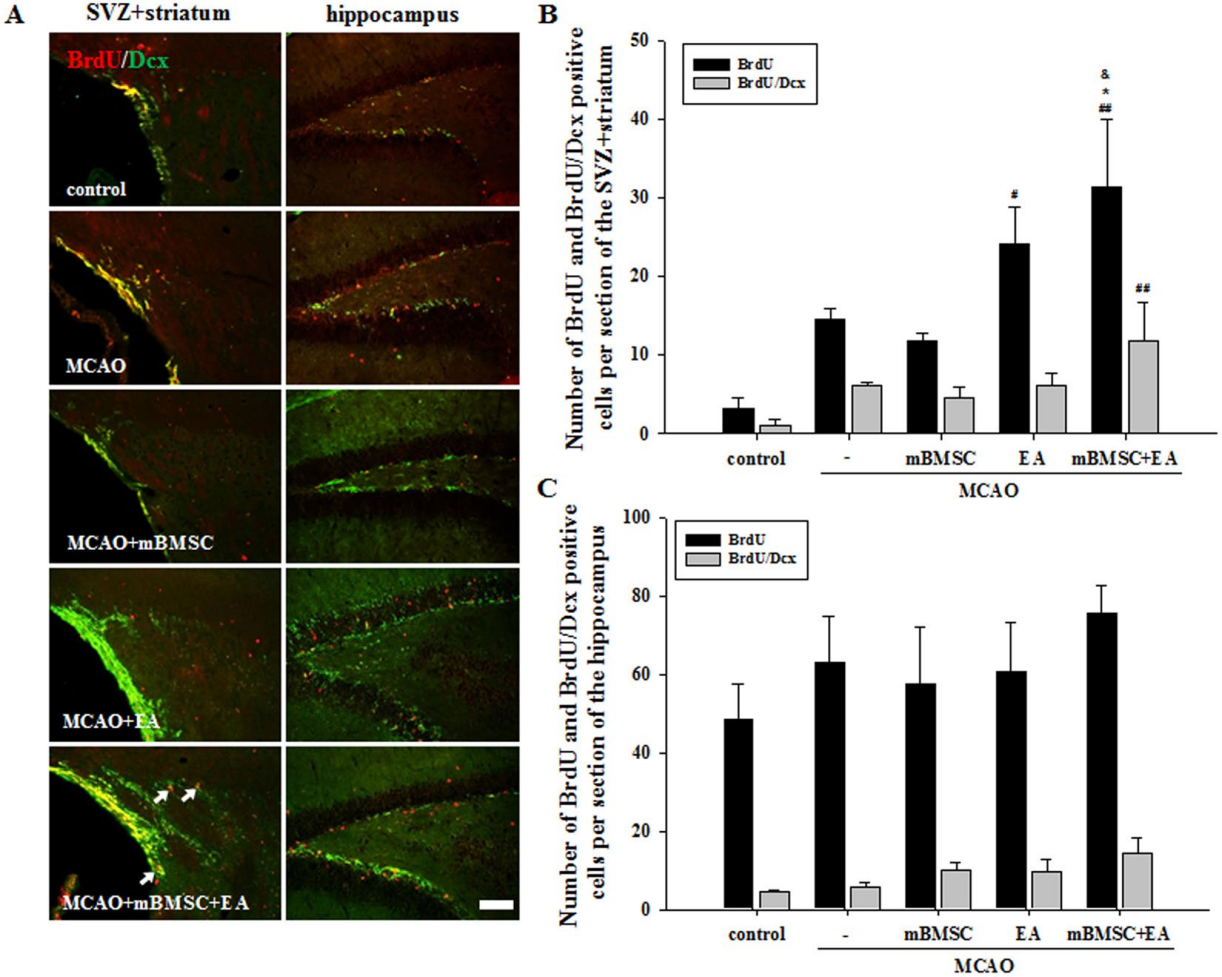
Effect of mBMSC and EA treatment on neurogenesis as detected by BrdU incorporation. (**A**) Photomicrographs and (**B**,**C**) histograms for BrdU-positive and BrdU/Dcx double-positive cells in the striatum and hippocampus (n = 5). The number of BrdU-positive cells in the striatum was significantly higher in the combined mBMSC and EA treatment group than in the other groups. BrdU/Dcx double-positive cells are indicated by arrows. ^#^*p* < 0.05 and ^##^*p* < 0.01 versus control group; ^*^p < 0.05 versus vehicle-treated MCAO group; ^&^*p* < 0.05 versus mBMSC-treated MCAO group. Scale bar = 100 μm.

**Figure 4:**
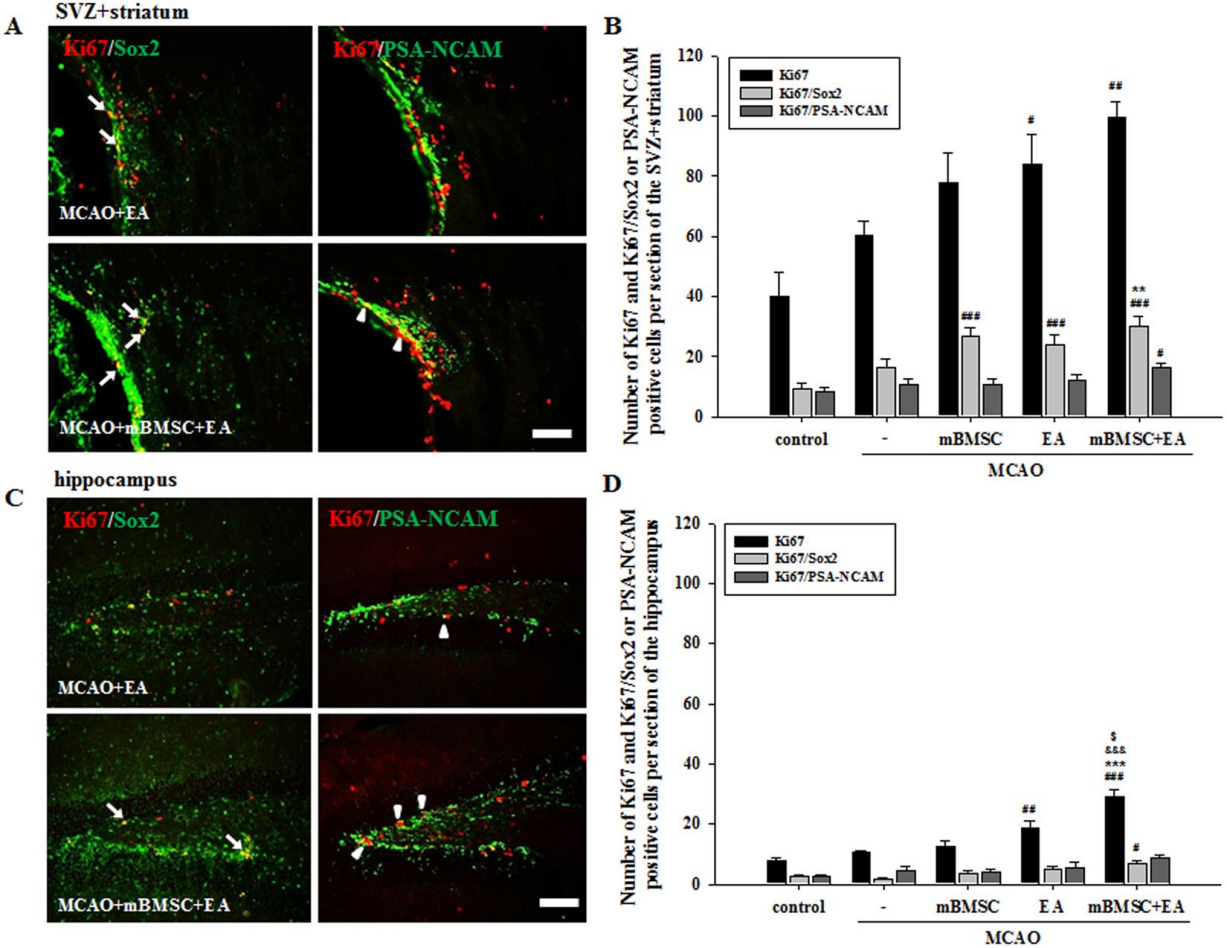
Effects of mBMSC and EA treatment on neurogenesis as detected by Ki67. (**A**,**C**) Photomicrographs and (**B**,**D**) histograms for Ki67-positive and Ki67/Sox2 or Ki67/PSA-NCAM double-positive cells (n = 5). The number of Ki67-positive cells, especially in the hippocampus, was higher in the combined mBMSC and EA treatment group than in the other groups. The number of Ki67/Sox2 double-positive cells in the striatum was significantly higher in the combined mBMSC and EA treatment group than in the vehicle-treated MCAO group. Ki67/Sox2 double-positive cells are indicated by arrows, and Ki67/PSA-NCAM double-positive cells by arrow heads. ^#^*p* < 0.05, ^##^*p* < 0.01, and ^###^*p* < 0.001 versus control group; ^**^p < 0.01 and ^***^p < 0.001 versus vehicle-treated MCAO group; ^&&&^*p* < 0.001 versus mBMSC-treated MCAO group. ^$^*p* < 0.01 versus EA-treated MCAO group. Scale bars = 100 μm.

The effects of mBMSC and EA on neurogenesis were analyzed in the subventricular zone (SVZ) with the surrounding area of the striatum and hippocampus of the ipsilateral hemisphere. In order to understand changes in neurogenesis as a result of mBMSC and EA treatment, the number of cells labeled with bromodeoxyuridine (BrdU) and neuronal marker doublecortin (Dcx) was analyzed (Fig. 3A). In the SVZ with the surrounding area of the striatum (SVZ + striatum), BrdU-positive cells were significantly higher in number in MCAO mice treated with a combination of mBMSC and EA than in mice treated with vehicle or mBMSC alone [F_(4,20)_ = 8.267, *p* < 0.001]. In addition, we found a higher number of BrdU/Dcx double-positive cells in the combination treatment group than in all other groups (Fig. 3B). In contrast, there was no difference in the number of labelled cells in the hippocampus (Fig. 3C). To confirm neurogenesis after mBMSC and EA treatment, the proliferation marker Ki67 was combined with a stem cell marker, sex-determining region Y-box 2 (Sox2), and an immature neuronal marker, polysialated neural cell adhesion molecule (PSA-NCAM) (Figs 4, S2, S3). The number of Ki67-positive cells in the SVZ + striatum and hippocampus was higher in the EA treatment group than in the control group. Moreover, we found a significant difference in the number of Ki67-positive cells, especially in the hippocampus, between the combined treatment group and all other MCAO groups [SVZ + striatum, F_(4,20)_ = 5.964, *p* = 0.003; hippocampus, F_(4,20)_ = 7.117, *p* = 0.001]. The number of Ki67/Sox2 double-positive cells in the SVZ + striatum was significantly higher in the combined treatment group than in the vehicle-treated MCAO group [F_(4,20)_ = 15.797, *p* < 0.001]. The number of Ki67/PSA-NCAM double-positive cells was significantly increased in the combined treatment group; the other treatment groups did not differ significantly from the control group (Fig. 4B,D). These results suggest that cell proliferation in the SVZ + striatum and hippocampus was significantly increased by treatment with a combination of mBMSC and EA, as we found a higher number of progenitor cells in this experimental group than in the vehicle-treated group.

### Effects of mBMSC and EA treatment on the expression of BDNF, NT4, and VEGF in the brain

**Figure 5:**
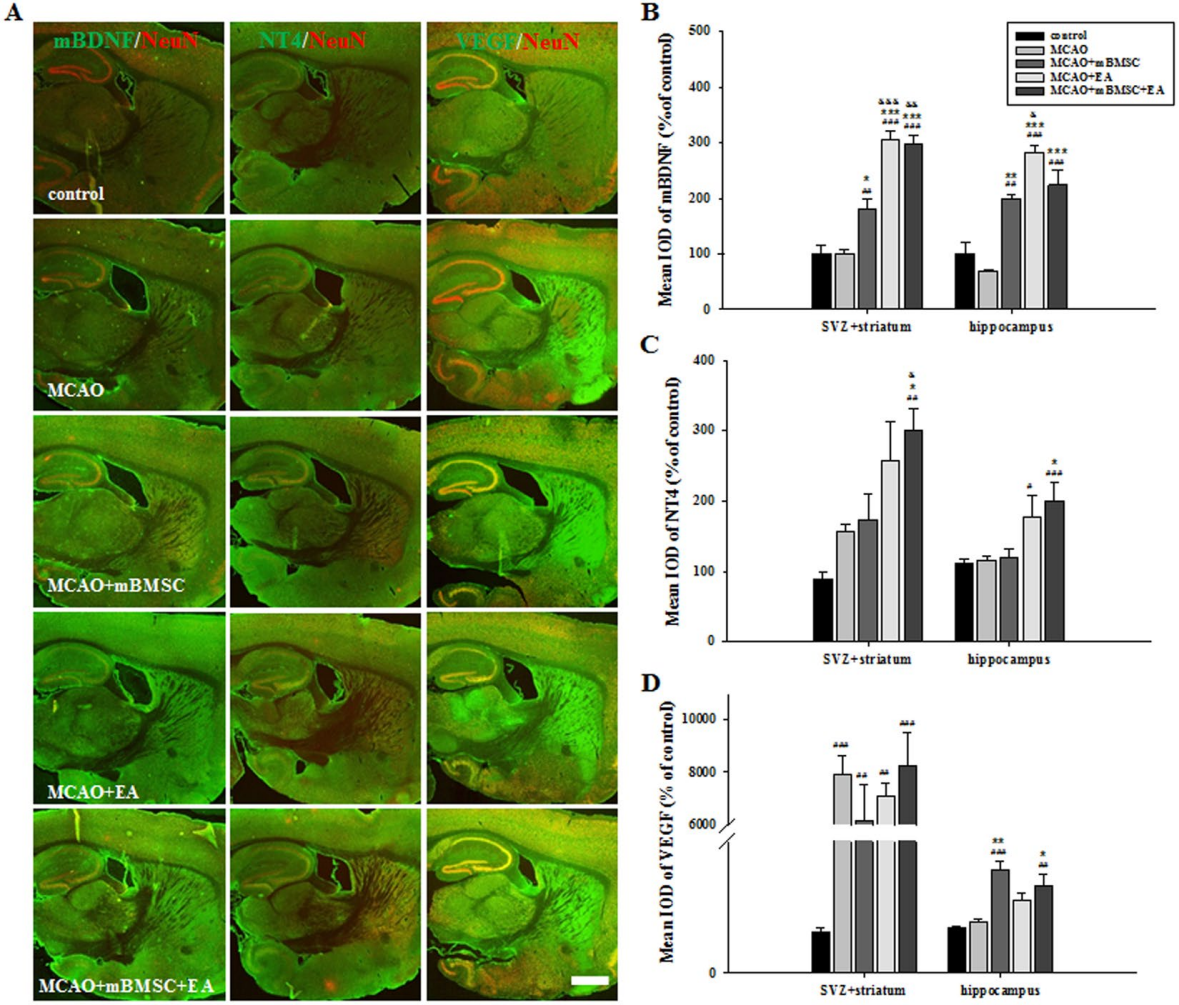
Effects of mBMSC and EA treatment on the expression of trophic factors. (**A**) Photomicrographs and (**B**‒**D**) histograms for mBDNF, NT4, and VEGF expression (n = 4–5). mBDNF expression was markedly increased by treatment with mBMSC and/or EA compared to vehicle treatment. mBDNF expression levels were higher in the EA-treated MCAO group than in the mBMSC-treated MCAO group. NT4 expression in the SVZ and hippocampus was significantly higher in the combined mBMSC and EA treatment group than in the other groups. VEGF expression in the hippocampus was markedly higher in the mBMSC treatment group than in the vehicle-treated MCAO group. ^#^*p* < 0.05, ^##^*p* < 0.01, and ^###^*p* < 0.001 versus control group; ^*^p < 0.05, ^**^p < 0.01, and ^***^p < 0.001 versus vehicle-treated MCAO group; ^&^*p* < 0.05, ^&&^*p* < 0.01, and ^&&&^*p* < 0.001 versus mBMSC-treated MCAO group; Scale bar = 1 mm.

Next, we investigated the effect of mBMSC and EA treatment on the expression of trophic factors affecting neurogenesis, such as brain-derived neurotrophic factor (BDNF) and neurotrophin-4 (NT4), and the vascular permeability factor, namely vascular endothelial growth factor (VEGF), in the SVZ with the surrounding area of the striatum and hippocampus of the ipsilateral hemisphere (Fig. 5A). The expression of mature BDNF (mBDNF) in the SVZ + striatum and hippocampus was significantly higher in the treatment groups (mBMSC alone, EA alone, mBMSC + EA) than in the vehicle-treated MCAO group. In particular, MCAO mice treated with EA had significantly different mBDNF levels than MCAO mice treated with mBMSC [Fig. 5B, SVZ + striatum, F_(4,20)_ = 42.714, *p* < 0.001; hippocampus, F_(4,20)_ = 25.770, *p* < 0.001]. The expression of NT4 in the SVZ + striatum and hippocampus was significantly higher in the combination treatment group than in the vehicle-treated MCAO group. Moreover, the combination treatment group showed a higher expression level of NT4 in the SVZ + striatum than the mBMSC-treated MCAO group [Fig. 5C, SVZ + striatum, F_(4,20)_ = 8.591, *p* < 0.001; hippocampus, F_(4,20)_ = 6.203, *p* = 0.004]. The expression of VEGF in the SVZ + striatum significantly increased following MCAO, compared to control mice without MCAO. There were no differences in the expression levels in the hippocampus among the treated groups, except for the mBMSC groups [Fig. 5D, SVZ + striatum, F_(4,18)_ = 11.445, *p* < 0.001; hippocampus, F_(4,18)_ = 9.828, *p* < 0.001].

**Figure 6:**
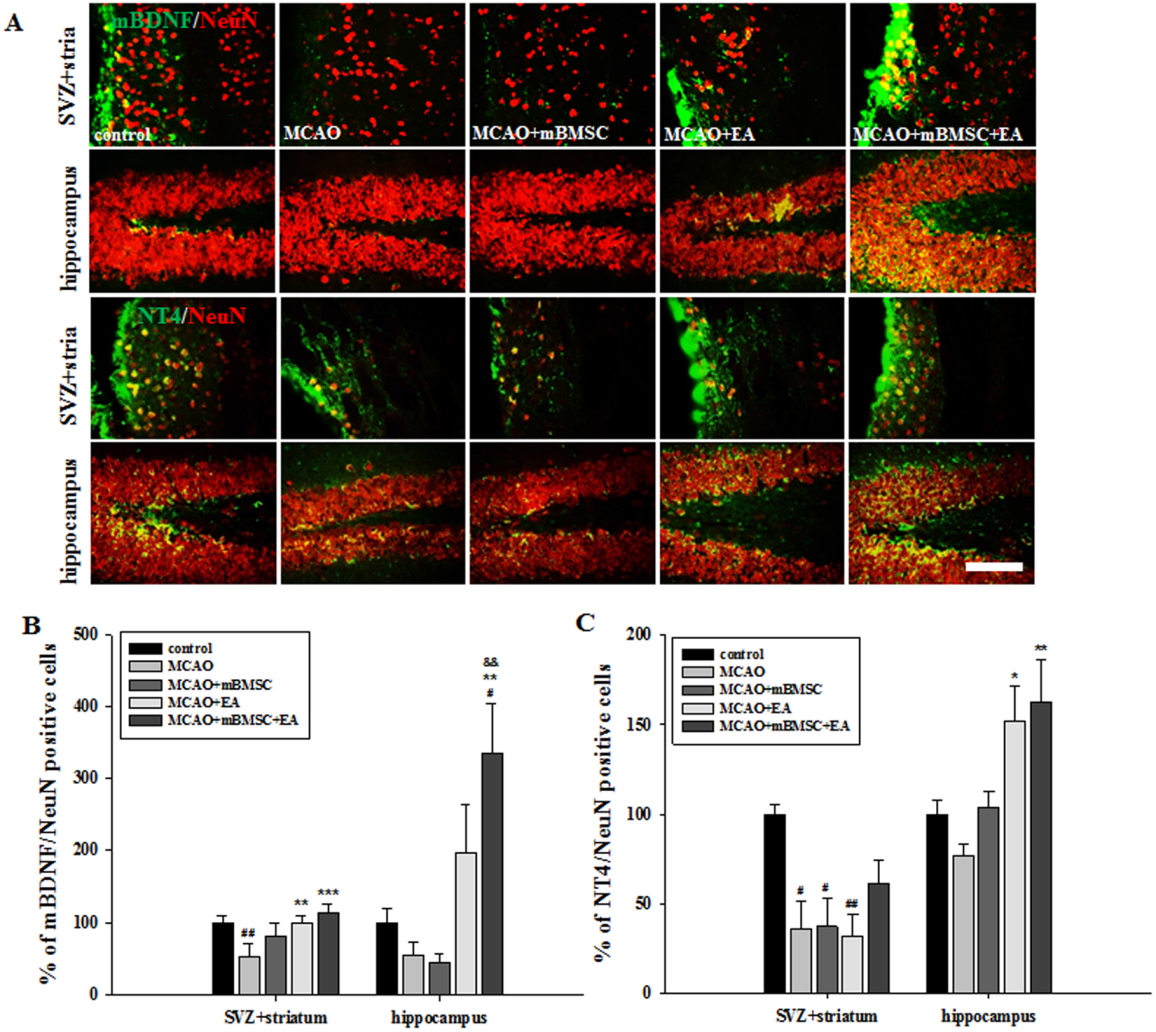
Effects of mBMSC and EA treatment on the expression of mBDNF and NT4 in neurons. (**A**) Photomicrographs and (**B**,**C**) histograms for mBDNF/NeuN or NT4/NeuN double-positive cells in the striatum and hippocampus (n = 5). The number of mBDNF/NeuN double-positive cells in the SVZ + striatum and hippocampus was significantly higher in the EA-treated MCAO group and the combined mBMSC + EA-treated MCAO group, compared to the number in the other groups. The number of NT4/NeuN double-positive cells in the hippocampus was significantly higher in the EA-treated MCAO group than in the vehicle-treated MCAO group. ^#^*p* < 0.05 and ^##^*p* < 0.01 versus control group; ^*^p < 0.05, ^**^p < 0.01, and ^***^p < 0.001 versus vehicle-treated MCAO group; ^&&^*p* < 0.01 versus mBMSC-treated MCAO group. Scale bar = 100 μm.

As shown in Fig. 5, we examined the changes in BDNF and NT4 expression levels in neuronal cells in the SVZ + striatum and hippocampus (Fig. 6A). The number of mBDNF/neuronal nuclei (NeuN) double-positive cells in the SVZ + striatum was significantly higher in the EA treatment group than in the vehicle group [F_(4,20)_ = 9.366, *p* < 0.001]. In the hippocampus, the number of mBDNF/NeuN double-positive cells in the combination treatment group was significantly higher than that in the vehicle or mBMSC treatment groups [Fig. 6B, F_(4,20)_ = 6.785, *p* = 0.001]. The number of NT4/NeuN double-positive cells in the SVZ + striatum was higher in the combination treatment group than in the other treatment groups [F_(4,20)_ 5.711, *p* = 0.005]. Mice treated with EA showed a higher number of these cells in the hippocampus than mice in the vehicle treatment group [Fig. 6C, F_(4,20)_ = 6.124, *p* = 0.003]. These results suggest that the beneficial effects of mBMSC and EA treatment were associated with changes in the expression levels of neurotrophic factors, mainly mBDNF and NT4, induced by the EA treatment.

### Effects of mBMSC and EA treatment on the activation of CREB in the brain

**Figure 7:**
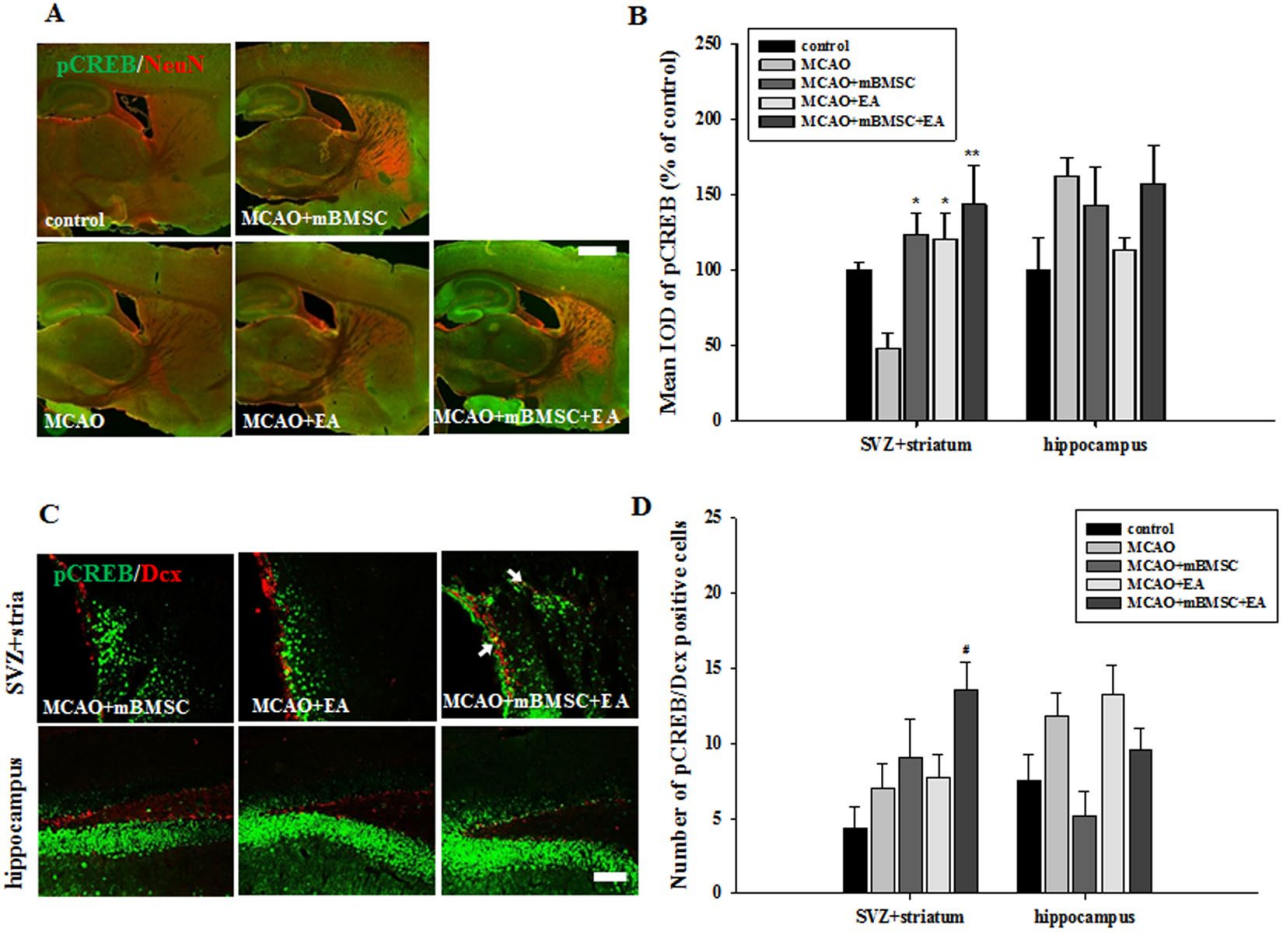
Effect of mBMSC and EA treatment on the expression of pCREB. (**A**,**C**) Photomicrographs and (**B**,**D**) histograms for pCREB and pCREB/Dcx (n = 5). pCREB expression in the striatum was markedly increased by treatment with mBMSC or EA. The number of pCREB/Dcx double-positive cells was significantly higher in the combination treatment MCAO group than in the control group. pCREB/Dcx double-positive cells are indicated by arrows. ^#^*p* < 0.05 versus control group; ^*^p < 0.05 and ^**^p < 0.01 versus vehicle-treated group. Scale bars = 1 mm (**A**) and 100 μm (**C**).

We found that the SVZ + striatum of MCAO mice treated with mBMSC and EA had higher levels of phosphorylated tropomyosin receptor kinase B (pTrkB), the common receptor of BDNF and NT4, than the SVZ + striatum of mice in the other groups, but this difference was not significant (data not shown). To investigate the effects of mBMSC and EA treatment on the activation of transcription factor cyclic AMP response element binding protein (CREB), we examined the expression of phosphorylated CREB (pCREB) in the same regions (Fig. 7). The pCREB levels in the SVZ + striatum were significantly higher in MCAO mice treated with mBMSC or EA than in mice in the vehicle group [F_(4,20)_ = 4.993, *p* = 0.007]. However, there was no significant difference in the hippocampus (Fig. 7A,B). The number of pCREB/Dcx double-positive cells in the SVZ + striatum was higher in the combination treatment group than in the vehicle-treated group [Fig. 7C,D, F_(4,20)_ = 3.592, *p* = 0.025]. These results suggest that treatment with mBMSC and EA induced the activation of the transcription factor CREB in the brain following ischemic stroke.

### Effects of mBMSC and EA treatment on the concentration of BDNF, NT4, and VEGF, and on the expression of pCREB in the brain

**Figure 8:**
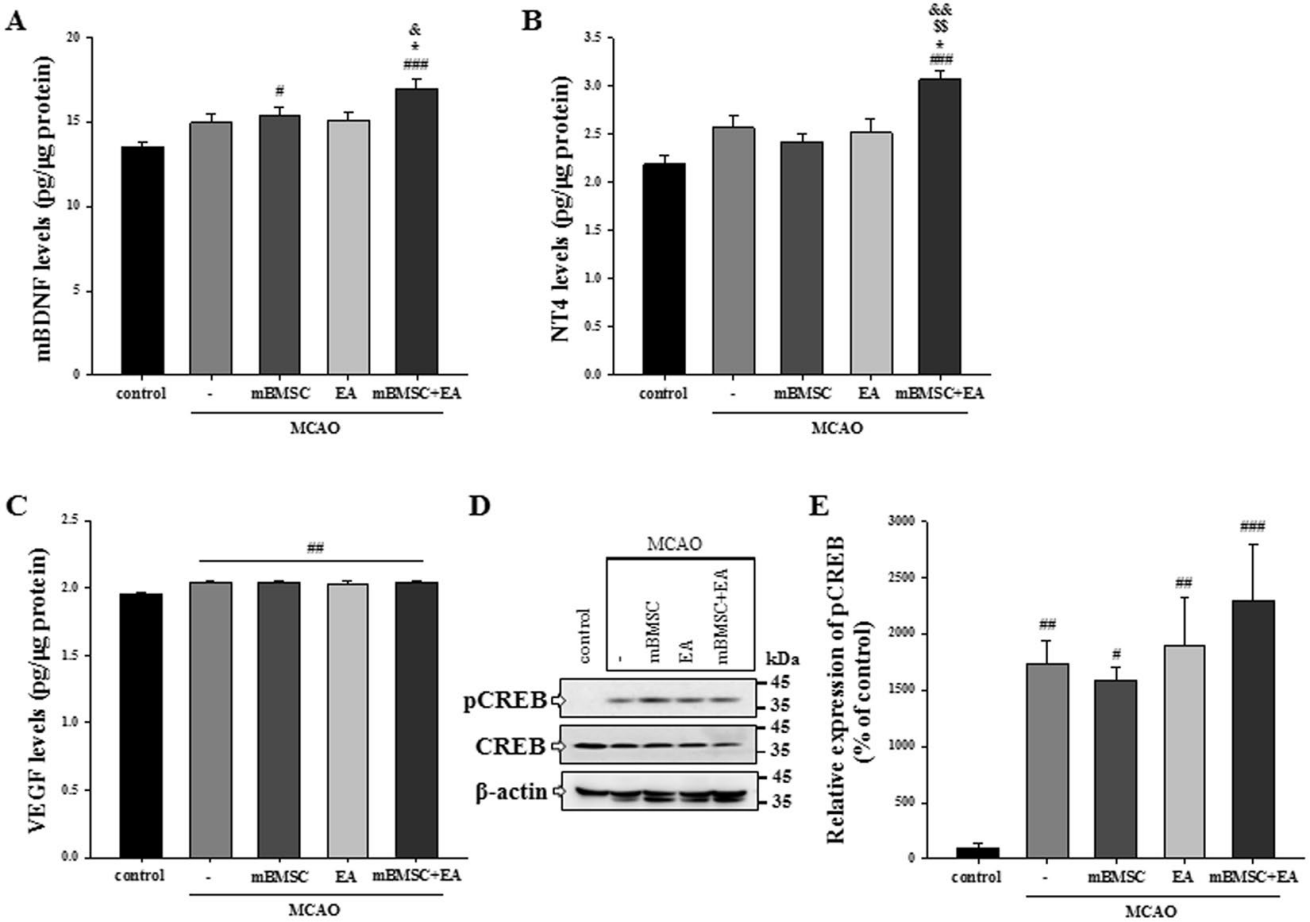
Effect of mBMSC and EA treatment on the concentration of BDNF, NT4, and VEGF and on the expression of pCREB in the ipsilateral brain. ELISA assays for (**A**) mBDNF, (**B**) NT4, and (**C**) VEGF (n = 9). Concentration levels of mBDNF and NT4 were significantly increased in the combination treatment group compared to the vehicle-treated MCAO group. ^#^*p* < 0.05, ^##^*p* < 0.01, and ^###^*p* < 0.001 versus control group; ^*^p < 0.05 versus vehicle-treated MCAO group; ^$$^*p* < 0.01 versus mBMSC-treated MCAO group; ^&^*p* < 0.05 and ^&&^*p* < 0.01 versus EA-treated MCAO group. (**D**) Western blot analysis and (**E**) its densitometry (n = 9). The expression of pCREB protein was also increased in the combination treatment group compared to the other MCAO-treated groups, but the difference was not significant. ^#^*p* < 0.05, ^##^*p* < 0.01, and ^###^*p* < 0.001 versus control group. Uncropped images of western blots are shown in Fig. S4.

To clarify whether mBMSC and EA treatment could influence the expression of trophic factors and the activation of pCREB in the brain, we confirmed the preceding results by ELISA tests and western blot analysis in the whole ipsilateral hemisphere of the brain including the SVZ + striatum and hippocampus. The ELISA results showed that levels of mBDNF were significantly higher in the combination treatment group than in the vehicle and EA-treated MCAO group [Fig. 8A, F_(4,40)_ = 7.284, *p* < 0.001]. Levels of NT4 were also significantly increased in the combination group compared to the other groups [Fig. 8B, F_(4,40)_ = 8.426, *p* < 0.001]. Levels of VEGF were significantly increased following MCAO compared to control mice, but there was no significant difference in the expression levels among all MCAO-treated groups [Fig. 8C, F_(4,40)_ = 5.676, *p* = 0.001]. Next, we confirmed the expression of pCREB in the same samples using western blot analysis. A significant increase in the expression of pCREB was observed in all MCAO-treated groups compared to the control group [Fig. 8D,E, F_(4,40)_ = 7.085, *p* < 0.001]. The combination treatment group showed higher expression than the other MCAO-treated groups, but the difference was not significant. These results suggest that treatment with mBMSC and EA might change the levels of trophic factors, mBDNF and NT4, and then stimulate CREB activation in the ipsilateral brain following ischemic stroke.

## Discussion

Although successful neurogenesis in the adult brain provides an attractive therapeutic prospect for treatment of brain injuries such as ischemic stroke^2,3^, only a limited number of mature cells have been found to provide cellular replacement^26–28^. Adult neurogenesis requires various extrinsic trophic factors to sustain and regulate the proliferation and differentiation of neuronal progenitor cells^3–5,29^. Thus, the activation of trophic factors in the brain can be an effective strategy for successful neurogenesis, as it can allow mature cells to develop from neuronal progenitor cells, thereby exerting a therapeutic effect on the ischemic brain.

MSC injection in immunodeficient as well as healthy animals promotes neurogenesis through elevated secretion of trophic factors such as BDNF, nerve growth factor (NGF), and VEGF^30,31^. MSCs also promote neurogenesis by stimulating synthesis factors in the adjacent cells^30^. Previous studies have shown that EA can affect the synthesis and secretion of various trophic factors in the central nervous system, which can mediate its therapeutic action on motor or cognitive function in animal models of ischemic stroke^6,20,22^. A systematic review of acupuncture studies suggests that acupuncture improves neurological deficits in ischemic stroke by enhancing endogenous neurogenesis^20^.

The results from our behavioral and histological studies show that treatment with a combination of mBMSC and EA restores the motor function of both paws and alleviates atrophic changes in the striatum, the main injury region of mice with MCAO. Further, EA treatment, and not mBMSC treatment, following MCAO improves motor function related to turning to the ipsilateral side. Thus, the effect of EA stimulation on neurological functional recovery in the ischemic brain is likely more effective than mBMSC transplantation.

Ischemic assault can usually stimulate neurogenesis in the adult brain^32^. Neurogenesis is characterized by increased proliferation of neural progenitor cells and migration of these cells towards the ischemic region^32,33^. However, the hostile environment in the ischemic region results in neuroblast death^34^. Thus, a limited number of cells that are positive for neuronal markers provide cellular replacement at the site of injury in the brain^8,27^. We assessed the effects of mBMSC and EA on neurogenesis following ischemic stroke using cell proliferation markers (BrdU and Ki67) and differentiation makers (Sox2, Dcx, and PS-NCAM) in the SVZ and surrounding striatum and hippocampus, the main sites associated with general neurogenesis.

We found that treatment with a combination of mBMSC and EA markedly promoted cell proliferation in the SVZ + striatum and hippocampus. The number of Ki67/Sox2 double-positive cells, which are known to be capable of differentiation, also significantly increased with the combination treatment. The number of cells positive for differentiation markers, such as BrdU/Dcx or Ki67/PSA-NCAM double-positive cells, in the SVZ + striatum was also higher in the combination treatment group than in the other treated groups, although this difference was not statistically significant. Previous studies have focused on the expression of trophic factors that enhance neurogenesis as one of the possible mechanisms underlying this effect. The ability of MSCs or EA to stimulate expression of trophic factors in the brain through autocrine or paracrine secretion may serve as a therapeutic approach for ischemic assault.

MSC transplantation is reported to promote the expression of trophic factors including BDNF, glial cell line-derived neurotrophic factor, NT3, and VEGF in the injured brain^18,35,36^. Expression of these factors is accompanied by enhanced cell proliferation and differentiation^18,36^. In particular, BDNF is considered to be a key factor underlying the trophic effects of MSC therapy^37^. EA treatment promotes neurogenesis or proliferation of reactive astrocytes that are associated with the enhanced expression of BDNF^38,39^. Moreover, treatment with a combination of EA and NGF has been shown to have a synergistic effect on functional recovery through cell proliferation and survival^40^. Our previous results showed that EA treatment promotes functional recovery following ischemic assault through the enhancement of proliferation and differentiation of neural stem cells via the BDNF, VEGF, and NT4 signaling pathways^24,25^.

We found in both immunohistochemical and ELISA assay results that mBMSC and EA treatment could lead to an increase in the expression levels of trophic factors such as BDNF, NT4, and VEGF, which are all important for brain tissue regeneration^41,42^. BDNF is essential for the development of the nervous system, and plays a role in proliferation, migration, differentiation, synaptic plasticity, and regulation of adult neurogenesis^3,43^. Our results suggest that mBMSC and EA can modulate neurotrophic factor secretion in the brain, especially that of BDNF, via the upregulation of endogenous neurogenesis. Especially EA led to significant differences in BDNF and NT4 expression when compared to mBMSC treatment, similar to the findings of previous studies^24,25^. VEGF is usually involved in inducing angiogenesis following ischemic injury, thereby providing neuroprotection to reduce the size of injury^44,45^. VEGF expression was significantly higher in the MCAO group, but there was no difference between the EA- and mBMSC-treated groups. It may be possible that we observe these expression levels due to the stimulation of secretion for self-recovery of the injured striatum site via angiogenesis following ischemic stroke.

The common binding target of BDNF and NT4, the TrkB receptor, plays a protective role in the pathophysiology of cerebral ischemia^3,46,47^. Neurotrophic factor-mediated TrkB signaling is important for the regulation of proliferation and enhanced neurogenesis of embryonic precursors, and for the maintenance of survival of neuronal populations^48,49^. TrkB signaling induces the production of the transcription factor, CREB, through the activation of protein kinase cascades; BDNF or NT4 expression coupled with CREB phosphorylation is thought to be one of the key modulatory mechanisms underlying neurogenesis^50–52^. We found that mBMSC and EA, especially EA, could influence the expression of neurotrophic factors such as BDNF and NT4, in turn stimulating the transactivation of CREB. This suggests that expression of both NT4 and BDNF interfering with the TrkB receptor has beneficial effects on ischemic injury^46^, and that the activation of CREB is an important regulatory signal within the neurogenic niche^50^. Combined EA and MSC treatment can promote the differentiation of oligodendrocyte-like cells and the formation of the myelin sheath via the NT3/TrkC signaling pathway^53^. Our findings also demonstrate the synergistic effects of mBMSC and EA in improving neurological motor function after ischemic injury by promoting neurogenesis via the activation of neurotrophic factors.

In summary, mBMSC and EA can activate the expression of neurotrophic factors such as BDNF and NT4, which are associated with neurogenesis in the ischemic brain. MSC transplantation combined with EA treatment, both stimulators of neurotrophic factors, can thus lead to the recovery of neurological function from ischemic injury. Our results provide data that corroborates the therapeutic benefits of treatment with a combination of mBMSC and EA, and suggest possible biological mechanisms through which this approach leads to an amelioration of neurological impairments by enhanced neurogenesis.

## Methods

### Animals

**Figure 9:**
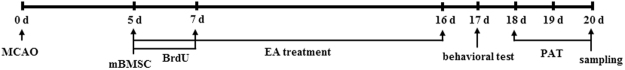
Schematic diagram of the experimental procedures. The time point of each experiment is described in relation to the day the MCAO was induced.

Male C57BL/6 and C57BL/6-Tg (CH-EGFP) mice purchased from Dooyeol Biotech (Seoul, Korea) were housed at 22 °C under an alternating 12 h light/dark cycle. All experiments were approved by the Pusan National University Animal Care and Use Committee, and the experimental procedures were in accordance with the National Institutes of Health Guidelines (approval number, PNU-2016-1117). The experimental schedule is schematically represented in Fig. 9.

### mBMSC isolation and culture

Male C57BL/6-Tg (CH-EGFP) mice (age: 5 weeks) were anesthetized with a 8% chloral hydrate solution, and the femur and tibia bones were removed. The bone marrow was obtained by injecting phosphate-buffered saline (PBS) into the tibia and the femur using a 10-ml syringe. On a clean laboratory bench, the bone marrow was filtered through a 70-µm cell strainer (Corning Inc., Corning, NY, USA) and washed twice with PBS. The bone marrow was then cultured on a 60-mm culture dish (SPL Life Sciences Co., Ltd., Pocheon, Korea) containing 20% fetal bovine serum (FBS; GE Healthcare Life Sciences, Logan, UT, USA), 1% penicillin/streptomycin (P/S; Thermo Fisher Scientific Inc., Waltham, MA, USA), and α-minimal Eagle’s medium (MEM; Thermo Fisher Scientific Inc.), and stored in a CO_2_ incubator (HEPA CLASS 100, Thermo Fisher Scientific Inc.) maintained at 37 °C. After 5 days, the medium was changed and the floating cells were discarded. The medium was replaced every 2–3 days, and the cells were subcultured using trypsin LE (Thermo Fisher Scientific Inc.) if the mBMSCs occupied approximately 80% of the dish.

### Characteristics of mBMSC

The characteristics of mBMSCs were confirmed by alkaline phosphatase, Sox2, and β-galactosidase staining (Fig. S1). We first used an alkaline phosphatase detection kit (SCR004, Millipore Corporation, Billerica, MA, USA), because alkaline phosphatase levels in stem cells are higher than in other cells. The mBMSCs that had been cultured for 5 days were fixed with 4% paraformaldehyde and stained with Naphthol/fast red violet solution. After 15 min, the stained mBMSCs were covered with 1X PBS and stem cell colonies were observed using an Olympus TH4–200 microscope (Olympus Corporation, Tokyo, Japan). For Sox2, which is essential for maintaining self-renewal of stem cells, samples were blocked with antibody dilution buffer (1X PBS containing 5% normal goat serum and 0.3% triton X-100). Then, samples were stained with Sox2 antibody (Cat. ab79351, Abcam, Cambridge, UK) at 4 °C overnight and incubated with the secondary antibodies for 2 h. To determine the degree of mBMSC senescence, we used a senescence β-galactosidase staining kit (# 9860, Cell Signaling Technology, Danvers, MA, USA). Cultured mBMSCs were fixed with 1X fixative solution and stained with β-galactosidase staining solution at 37 °C (no CO_2_). The cells were overlaid with 70% glycerol and observed. The degree of mBMSC senescence was confirmed using an Olympus TH4–200 microscope (Olympus Corporation). Only mBMSCs obtained at passage 3 were used for transplantation.

### MCAO model

Male C57BL/6 J mice (age: 8 weeks) were anesthetized with 2% isoflurane in 20% O_2_ and 80% N_2_O using a model VIP 3000 calibrated vaporizer (Midmark, Orchard Park, OH, USA). Body temperature was maintained at 37 °C during the operation using a heating pad. Cerebral blood flow was measured by attaching a PeriFlux Laser Doppler System 5000 (Perimed, Stockholm, Sweden) to the skull of the mice. Focal cerebral ischemia was induced in the middle cerebral artery through the internal carotid artery by inserting the filament (Doccol Corporation, Sharon, MA 02067–2427, USA) into the common carotid artery. The inserted filament was removed and reperfused after 40 min.

### mBMSC transplantation

Male C57BL/6 J mice (age: 8 weeks) were anesthetized with 2% isoflurane in 20% O_2_ and 80% N_2_O using a model VIP 3000 calibrated vaporizer (Midmark). The mBMSCs from passage 3 were prepared at a concentration of 1X 10^5^/5 µl, and these mBMSCs (or PBS) were stereotaxically transplanted into the left striatum (ML: 2.5 mm, DV: 3.5 mm) at a speed of 0.5 µl/min using a 26-gauge Hamilton syringe (Hamilton Company, Reno, NV, USA). The syringe was removed from the brain 2 min after the mBMSCs were fully inserted into the striatum.

### EA treatment

Mice were anesthetized with 2% isoflurane containing 80% N_2_O and 20% O_2_ on a heating pad maintained at 37 °C. Stainless steel needles (Cat. DB106, Dongbang Healthcare Co., Ltd., Seoul, Korea) were inserted into the acupoints, namely the Baihui (GV20, midpoint of the line connecting the apexes of the two ears on the parietal bone) and Dazhui (GV14, posterior midline in the depression below the spinous process of the 7th cervical vertebra) points. The needle was connected to the Grass S88 electrostimulator (Astro-Med Inc., West Warwick, RI, USA), and the acupoints were stimulated (2 Hz, 2 V) for 20 min. EA was performed once a day for 12 days from day 5 to day 16 after MCAO. The control, MCAO, and MCAO + mBMSC groups were anesthetized with isoflurane without EA stimulation.

### BrdU injection

Injection of bromodeoxyuridine, a synthetic nucleoside (BrdU; B5002–5G; Sigma-Aldrich, Merck KGaA, Darmstadt, Germany), is a method of labeling cells to observe the extent of proliferation. BrdU (10 mg/ml; 50 mg/kg of bodyweight) was administered once a day from day 5 to day 7, the most active period of cell proliferation, after MCAO.

### Behavioral tests

Corner and cylinder tests were performed on day 17 after MCAO, and the passive avoidance test was performed on days 18–20 after MCAO.

#### Corner test

The corner test is a behavioral test used to assess sensorimotor dysfunction on the ipsilateral side following ischemic stroke. Mice that have suffered brain damage by MCAO have functional deficits on the contralateral side (i.e., the paw opposite to the damaged brain hemisphere) due to nerve crossover. These mice use their ipsilateral (unaffected) paws more often to turn at a corner. To test mice on this characteristic in the current study, two boards (size: 30 cm × 20 cm) were attached to each other at an angle of 30°, with one side left open. The direction in which the body of the mouse was raised at the inside corner was assessed. This procedure was repeated 10 times, and the number of turns to the ipsilateral side was calculated as a percentage.

#### Cylinder test

The cylinder test is a method used for evaluating sensorimotor function by confirming the use of the forelimb. When the mouse enters a transparent acrylic cylinder (9 cm × 9 cm × 15 cm), it touches the front wall of the cylindrical wall. The number of contacts made with the right paw, the left paw, and both paws over the course of 20 trials was calculated. The degree of use of each paw was then expressed as a percentage.

#### Passive avoidance test

The passive avoidance test was conducted to verify short-term learning and memory. This test was based on the fact that mice avoid bright light. Mice were placed in bright light and, when they tried to escape from the light, experienced a foot shock. Their memory of the foot shock was confirmed the following day. The mice underwent training for this task on day 18 after MCAO, and the task was then repeated on the following three days. The passive avoidance device (MED-APA-D1, Med Associates Inc., St. Albans, VT, USA) had two compartments: a bright and a dark box. When the required conditions for the experiment were entered into the program, the guillotine door automatically opened and closed. The mice were placed in the bright compartment, and entered the dark compartment for a period of 180 s, during which the guillotine door was closed and the mice received a mild electric shock (0.1 mA, 3 s). On the next day, the latency time for transfer from the bright compartment to the dark compartment was recorded. The compartment was cleaned with 70% ethanol solution before each experiment.

### Cresyl violet staining

Mice were intraperitoneally anesthetized with 8% chloral hydrate, and perfused with PBS containing 4% paraformaldehyde. Their brains were dissected and post-fixed in 4% paraformaldehyde. After three days in 30% sucrose solution, the samples were embedded into an optimum cutting temperature compound and stored at −80 °C. Next, the samples were cryo-sectioned at −20 °C to obtain 25-μm-thick sagittal sections. These sections were placed on a glass slide and stained with cresyl violet (Sigma-Aldrich Corporation, Saint Louis, Mo., USA) solution for 2 min. Next, the samples were serially dehydrated in alcohol and cleared in xylene for 20 min. Images were obtained using optical microscopy (Stemi 305, Carl Zeiss AG, Oberkochen, Germany), and brain atrophy was analyzed by volumetric analysis using the i-solution program (IMT i-Solution Inc., Burnaby, BC, Canada).

### Immunohistochemical analysis

For immunohistochemical analysis, 25-μm-thick sagittal serial cryosections (2.0–3.0 mm from the midline) of the ipsilateral hemisphere were obtained. The samples were post-fixed with 4% paraformaldehyde solution for 15 min and then washed with PBS. Next, the samples were blocked with antibody dilution buffer (1X PBS containing 5% normal goat serum and 0.3% triton X-100) and then treated using the general immunofluorescence protocol for cell signaling assays. They were stained with the following primary antibodies at 4 °C overnight: BrdU (Cat. OBT0030G, AbD Serotec, Oxford, UK), Dcx (Cat. Ab18723, Abcam, Cambridge, UK), Ki67 (Cat. Ab15580, Abcam), NeuN (Cat. MAB377; ABN78, Millipore Corporation, Billerica, MA, USA), mature BDNF (Cat. NB100–98682, Novus Biologicals, LLC, Littleton CO, USA), NT4 (Cat. sc-545, Santa Cruz Biotechnology, Santa Cruz, CA, USA), pCREB (Cat. sc-7978-R, Santa Cruz Biotechnology), PSA-NCAM (Cat. MAB5324, Millipore Corporation), pTrkB (phospho Y515, Cat. ab131483, Abcam), Sox2 (Cat. ab79351, Abcam), and VEGF (Cat. MAB360, Millipore Corporation). The next day, the samples were washed with PBST and incubated with the secondary antibodies for 2 h. The stained brain samples were covered with mounting solution with DAPI (H-1200, Vector Laboratories, Inc., Burlingame, CA, USA). For the cell counting, we selected five brain slices and captured images of sagittal brain sections at 20× magnification. The cell number and integrated optical density (IOD) were measured using i-solution (IMT i-solution Inc.) after acquiring images using fluorescence (Carl Zeiss Imager M1, Carl Zeiss AG) and confocal laser scanning (LSM 510, Carl Zeiss AG) microscopes.

### Measurement of mBDNF, NT4, and VEGF concentrations

Tissue samples of the ipsilateral hemisphere were homogenized with lysis buffer containing 200 mM Tris (pH 8.0), 150 mM NaCl, 2 mM EDTA, 1 mM NaF, 1% NP40, 1 mM PMSF, 1 mM Na_3_VO_4_, and a protease inhibitor cocktail, and then centrifuged at 13,000 rpm for 30 min at 4 °C. Levels of mBDNF, NT4, and VEGF were measured in the tissue supernatant samples using a Quantikine BDNF ELISA kit (Cat. DBD00, R&D Systems Inc., Minneapolis, MN, USA), an NT-4 DuoSet ELISA kit (Cat. DY268, R&D Systems Inc.), and a Quantikine VEGF ELISA kit (Cat. MMV00, R&D Systems Inc.). Final reactions were measured using a SpectraMax 190 microplate reader (Molecular Devices, Sunnyvale, CA, USA), and the concentrations of mBDNF, NT4, and VEGF were determined based on a standard curve according to the manufacturer’s instructions.

### Western blot analysis

Equal amounts of proteins in the supernatant of the ipsilateral hemisphere were separated on a 10% sodium dodecyl sulfate-polyacrylamide gel using electrophoresis (SDS-PAGE) and then transferred to a nitrocellulose membrane (Whatman GmbH, Dassel, Germany). Membranes were blocked with 5% non-fat dry milk dissolved in PBST for 1 h. After washing, the membrane was probed with primary antibodies against CREB (Cat. 9197, Santa Cruz Biotechnology), pCREB (Ser133, Cat. 9196, Cell Signaling), or actin (Cat. A2066, Sigma-Aldrich), overnight at 4 °C on a shaker. Membranes were then incubated with the appropriate horseradish peroxidase-conjugated secondary antibodies for 1 h. All specific bands were visualized using an enhanced chemiluminescence system (Pierce Biotech, Rockford, IL, USA) and imaged using an Image Quant LAS-4000 imaging system (GE Healthcare Life Science, Uppsala, Sweden).

### Statistical Analyses

The data were analyzed using the SigmaPlot statistical package version 11.2 (Systat Software, San Jose, CA, USA) and expressed as mean ± standard error of the mean (SEM). Group comparisons were made using one-way or one-way repeated-measures analysis of variance (ANOVA), followed by Tukey’s post-hoc test when comparing more than two groups. Results with a level of *p* < 0.05 were considered to be statistically significant.

## Electronic supplementary material

Supplementary Information
