# Supplementary material for: Potential benefits of mesenchymal stem cells and electroacupuncture on the trophic factors associated with neurogenesis in mice with ischemic stroke
Source: Sci Rep. 2018 Feb 1;8:2044. doi: 10.1038/s41598-018-20481-3 (PMC5794924; doi:10.1038/s41598-018-20481-3)
Supplement: Supplementary file 1 — Supplementary Information [file 41598_2018_20481_MOESM1_ESM.pdf]

Supplementary Information

Potential benefits of mesenchymal stem cells and electroacupuncture on the trophic factors associated with neurogenesis in mice with ischemic stroke

Yu Ri Kim[1,2], Sung Min Ahn[2], Malk Eun Pak[1,3], Hong Ju Lee[1,3], Da Hee Jung[1,3], Yong-Il Shin[4], Hwa Kyoung Shin[1,2,3,5], Byung Tae Choi[1,2,3,5*]

[1]Department of Korean Medical Science, School of Korean Medicine, Pusan National University, Yangsan 50612, Republic of Korea

[2]Korean Medical Science Research Center for Healthy Aging, Pusan National University, Yangsan 50612, Republic of Korea

[3]Graduate Training Program of Korean Medicine for Healthy Aging, School of Korean Medicine, Pusan National University, Yangsan 50612, Republic of Korea

[4]Department of Rehabilitation Medicine, School of Medicine, Pusan National University, Yangsan 50612, Republic of Korea

[5]Division of Meridian and Structural Medicine, School of Korean Medicine, Pusan National University, Yangsan 50612, Republic of Korea

*Correspondence and requests for materials should be addressed to B.T.C. (email: choibt@pusan.ac.kr)

Supplementary Information Figures

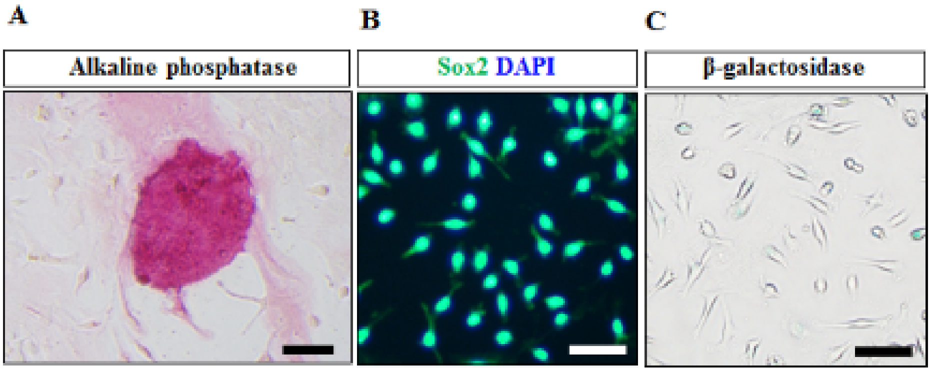

Fig. S1. Assessment of alkaline phosphatase, SOX2, and β-galactosidase activity in cultured mBMSCs obtained at passage 3. Phenotypic characteristics of stem cells were confirmed by (A) alkaline phosphatase and (B) SOX2 staining in cultured mBMSCs. (C) Senescence-associated β-galactosidase staining shown by blue granules was hardly seen in most mBMSCs. Scale bars=50 μm.

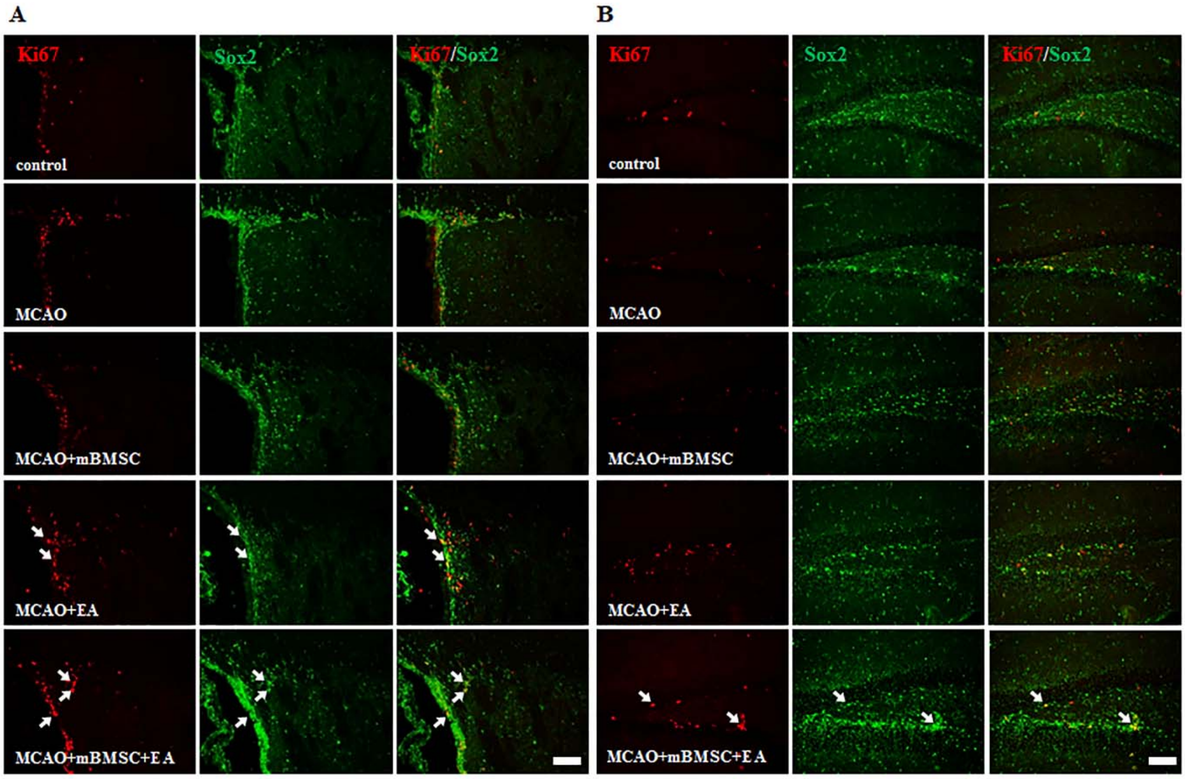

Fig. S2. Effects of mBMSC and EA treatment on proliferation marker as detected by Ki67 and Sox2. (A) Photomicrographs for Ki67 and Sox2 in the SVZ+striatum. (B) Photomicrographs for Ki67 and Sox2 in the hippocampus. Ki67/Sox2 double-positive cells are indicated by arrows. Scale bars=100 μm.

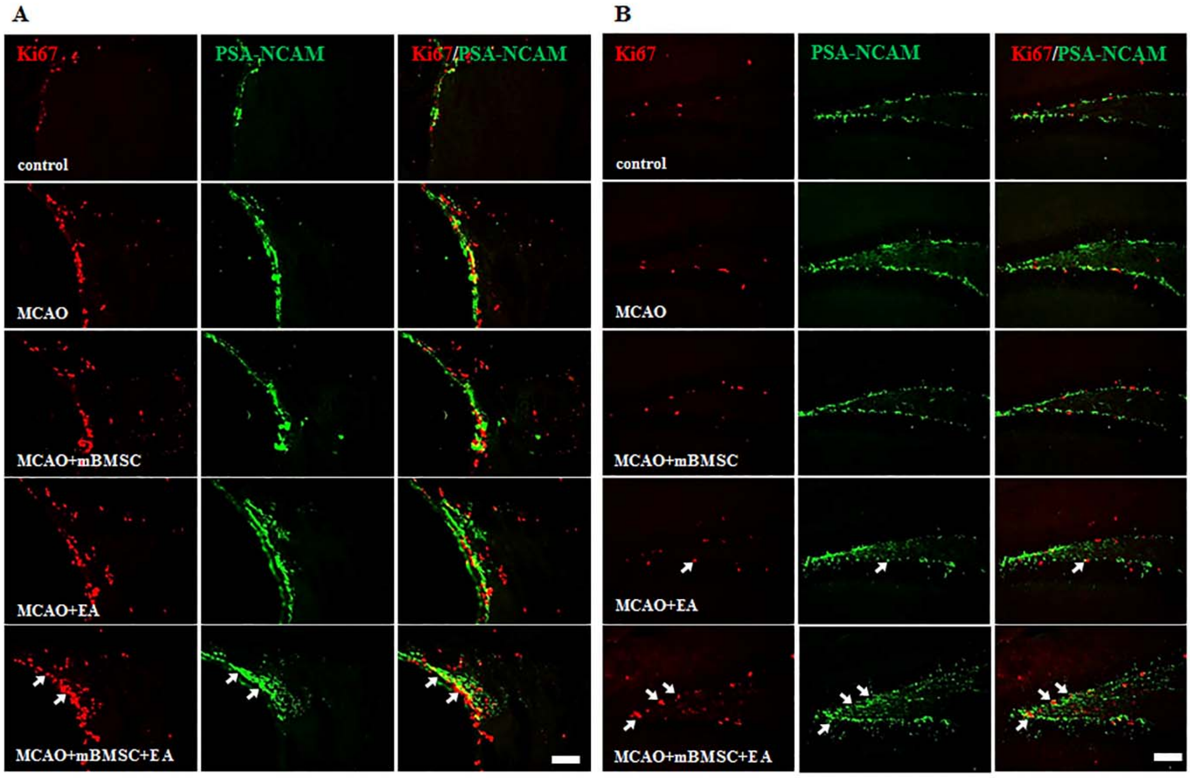

Fig. S3. Effects of mBMSC and EA treatment on immature neuronal marker as detected by Ki67 and PSA-NCAM. (A) Photomicrographs for Ki67 and PSA-NCAM in the SVZ+striatum. (B) Photomicrographs for Ki67 and PSA-NCAM in the hippocampus. Ki67/PSA-NCAM double-positive cells are indicated by arrows. Scale bars=100 μm.

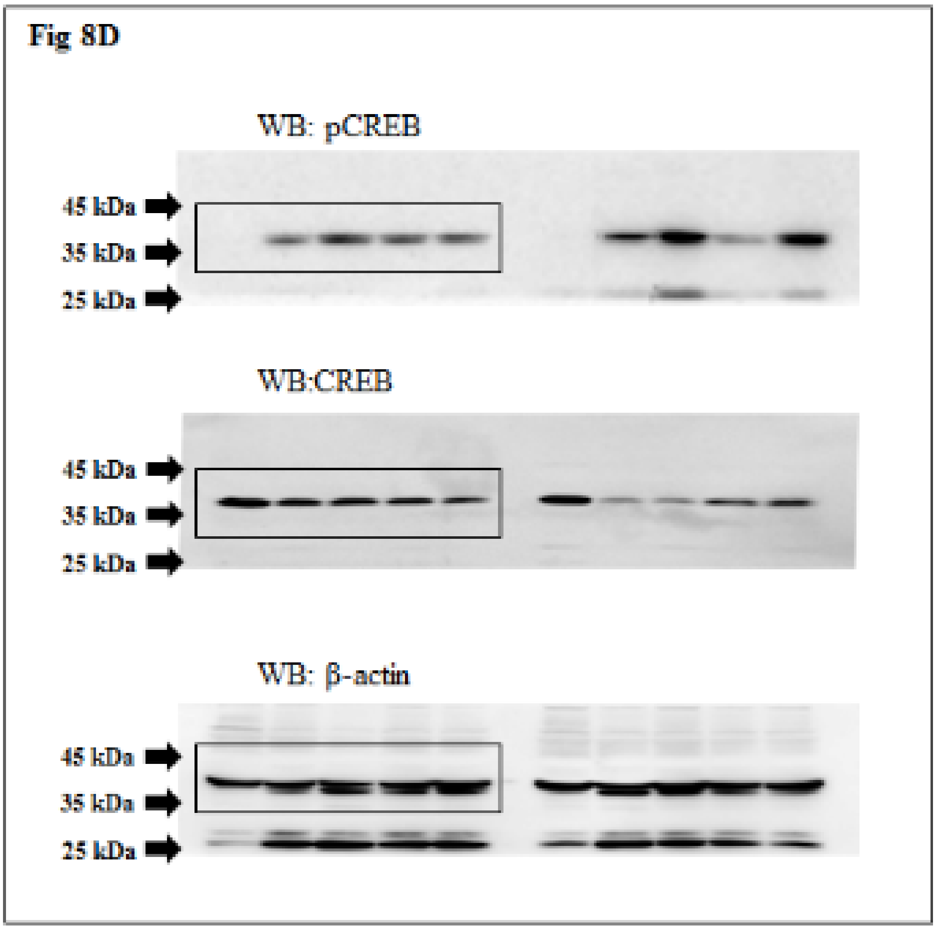

Fig. S4. Blot images of approximate regions used for figures marked with rectangles and molecular size marker (kDa).
